# Using the contributor role taxonomy (CRediT) as a tool in resolving authorship disputes at the NIH

**DOI:** 10.1080/08989621.2025.2596063

**Published:** 2025-12-07

**Authors:** Kathryn Partin, Mohammad Hosseini

**Affiliations:** a Office of Intramural Research, National Institutes of Health, Bethesda, USA; b Department of Preventive Medicine, Northwestern University Feinberg School of Medicine, Chicago, IL, USA; c Galter Health Sciences Library and Learning Center, Northwestern University Feinberg School of Medicine, Chicago, IL, USA

**Keywords:** Authorship, Publication Ethics, Authorship Disputes, CRediT Taxonomy, Research Integrity

## Abstract

The Contributor Role Taxonomy (CRediT) was released in 2014 with the aim of improving the attribution of credit and responsibilities in scholarly publications. Besides encouraging researchers to use CRediT for specification of contributions in publications, the National Institutes of Health (NIH) Intramural Research Program (IRP) has been using CRediT as a tool to investigate and resolve authorship disputes pre- and post-publication. In this article, we share the policies and procedures used at the NIH IRP for resolving authorship disputes, with the hope that other administrators and institutions might find value in our approach and provide feedback where necessary. The NIH IRP employs CRediT to offer a more objective and structured approach to understanding how a supervisor, complainant, or other parties involved in a dispute view the overall contributions in a project. This approach provides both the research group and the mediator or investigator with a common vocabulary to describe contributions and minimizes the likelihood of misunderstanding. Developing robust and transparent institutional mechanisms to address and resolve disputes, including guidance on how to address conflicts on authorship and authorship order, might contribute to a more productive and healthier research environment.

## Introduction

With some exceptions (e.g., conference proceedings in computer science and engineering education), peer-reviewed publications are the most coveted form of knowledge dissemination in science (Shamoo and Resnik 2022). There are several reasons why this is the case. First, naming a person as an author of a peer-reviewed manuscript is the primary means of recognizing their contribution to a research project, thereby giving them credit for their work, and holding them to account for the accuracy and reliability of their contributions (Shamoo and Resnik 2022). Second, research productivity metrics used by many universities to evaluate faculty for tenure and promotion are mostly based on citations in peer-reviewed publications (Berkwits 2024). Third, peer-reviewed publications and citations are significant factors in university rankings (Hosseini et al. 2022). Fourth, since peer-reviewed publications usually undergo a rigorous screening process in which other disinterested experts check the work’s accuracy and reliability, they are regarded as authoritative sources of knowledge (Flanagin 2024).

Due to these considerations, stakes are high in authorship, and disputes about different aspects of it are fairly common. These disputes involve controversies about who is named as an author, the order of authorship, and sometimes, who is named as the corresponding author (Smith et al. 2020). In biomedical research, first and last author are the most significant and sought after positions. The first author is the individual who contributed most significantly to the research, for example, to the conduct of investigations and wrote the first draft of the manuscript. Being named as a first author is so important that this position is sometimes shared among two or three coauthors – the so-called equal co-authorship. Typically, trainees, including undergraduate and graduate students, postdoctoral and clinical fellows, are first authors. The last author is usually the most senior or the principal investigator who led the project conceptualization, funding acquisition, methodology development, experimental design, and interpretation of the results, and also the individual who supervised the research team, including the first author (Bird, Hosseini, and Plemmons 2023).

However, because research is increasingly multidisciplinary, multi-site and international, multiple researchers might contribute significantly to the same tasks, or various contributors might be involved for different durations and to varying degrees. In such cases, determining who qualifies as an author, and, in what order authors should be listed can be extremely challenging. Authorship disagreements contribute to tension and contentious conflicts, which can slow down the productivity of research teams and negatively affect future collaborations (Bird, Hosseini, and Plemmons 2023).

## Authorship disputes

Authorship disputes are common and multifaceted. In a survey of active researchers (*n* = 4267) conducted by Wellcome Trust, “40% of all responded said that they had experienced issues with others taking credit for their work” (2020). In a mixed method study conducted by Bird, Hosseini, and Plemmons (2023) involving senior and junior researchers based in the US, China, Brazil, and Germany, 10 specific causes of confusion and controversy that result in authorship disputes were identified: 1) Varying moral norms and values in international contexts; 2) Internationalization and increased number of authors per publication; 3) Collaboration between junior and senior researchers; 4) Narrow and broad consideration of contributions; 5) Multidisciplinary perspectives; 6) Assumptions and over generalizations; 7) National and international issues; 8) Idiosyncrasies and individual differences; 9) Poor communication; and 10) Unpredictability of research.

Among these, collaboration between junior and senior researchers is a fertile ground for tension and one that is frequently observed. Due to involved power disparities in relationships, quite often, junior researchers who feel they are treated *unfairly* (e.g., the authorship order does not reflect the extent of my contributions, or I should be a co-author instead of that senior investigator), find it difficult to raise concerns or advocate for themselves. (Bird, Hosseini, and Plemmons 2023). For these reasons, many academic institutions have developed policies and procedures for assigning authorship and resolving disputes. Having institutional processes that fairly and consistently help resolve authorship disputes is generally viewed as a trait of a healthy research environment (Lauer 2023). In this article, we will describe the policies and procedures used at the National Institutes of Health (NIH) Intramural Research Program (IRP) with the hope that other researchers and institutions might learn from the NIH IRP’s approach and experiences with regard to resolving authorship disputes.

## Authorship dispute resolution at the NIH IRP

The NIH IRP, described in Schor (2024), performs investigator-initiated research similar to academic research institutions. Within this environment, the faculty-led Committee on Scientific Conduct and Ethics (CSCE) plays an active role in the design and implementation of the NIH IRP Responsible Conduct of Research (RCR) program. CSCE also drafts institutional guidance for the IRP, such as the Guidance and Policies for the Conduct of Research (Committee on Scientific Conduct and Ethics – CSCE 2023a), which provides ground rules for authorship and publication practices. Based on this guidance:

“Authorship on manuscripts or presentations serves two critical purposes in research: 1) to give credit for scientific discoveries and innovations and 2) to ensure accountability. While authorship can benefit individuals by helping them achieve recognition and advance their careers, it also implies responsibilities for the data and analyses, including sharing underlying materials, methods, and datasets with the scientific community. NIH policy supports the fair and responsible assignment of authorship to publications or presentations. Authorship should be based on the following:

1. Making a significant contribution to the conceptualization, design, execution, or interpretation of the research. AND
2. Drafting, revising, or carefully reading and confirming the research manuscript or presentation. AND
3. Taking responsibility for the research, particularly your contribution to it.

Individuals who meet the first and third criteria listed above must be allowed to read the manuscript or presentation so that they can meet all three criteria. Individuals who do not meet all three criteria should be acknowledged in the text, not in the author list. Individuals might be acknowledged for performing activities, such as providing encouragement, critical feedback, space, financial support, reagents, systems support, routine analyses, or patient material.”

**Figure 1.**
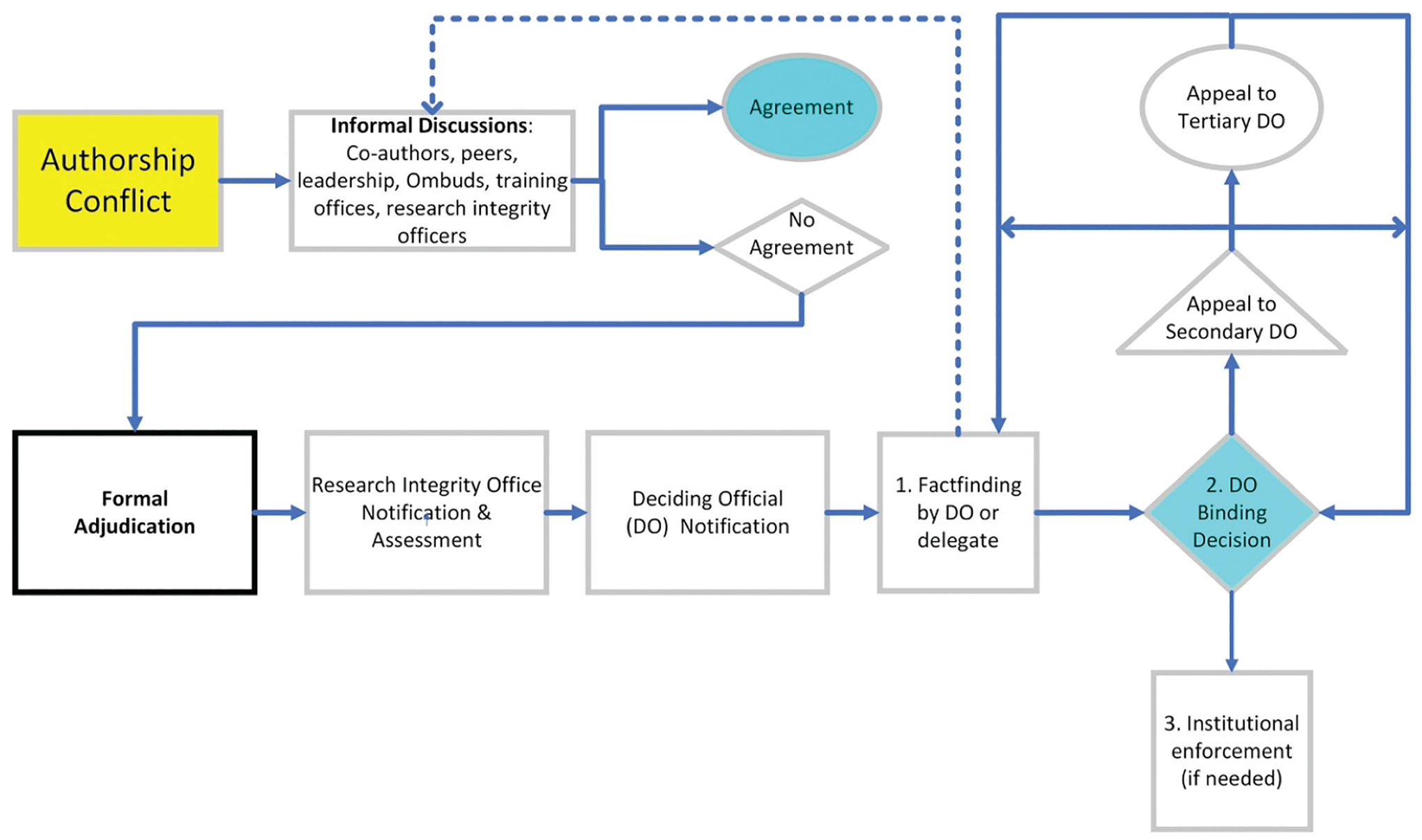
Representation of workflow through the informal and formal processes of authorship conflict resolution. If informal discussions fail to yield an agreement, any party to the conflict may trigger the formal adjudication process by sending an e-mail to the institutional research integrity officer. Once notified, the deciding official (DO) 1) performs factfinding, 2) renders a binding decision, and 3) ensures institutional compliance with the binding decision. Parties to conflict may appeal a determination to a secondary DO (usually the Deputy Director for Intramural research) and a tertiary DO (the Principal Deputy Director of NIH). Their determinations result either in execution of a binding decision or a return for additional factfinding.

Furthermore, the CSCE has developed and implemented the NIH IRP Authorship Conflict Resolution Policy, which was revised in 2023 (Committee on Scientific Conduct and Ethics – CSCE 2023b). The goal of this Policy is to offer a process that settles disputes “fairly, collegially, effectively, and expeditiously” (Committee on Scientific Conduct and Ethics – CSCE 2023b Para. 4). The Policy breaks the process into two stages: an informal dispute resolution stage, which, if unsuccessful, is followed by a formal adjudication stage (Figure 1).

### The informal dispute resolution stage

This stage reflects decades of established approaches to resolving conflicts among researchers and has traditionally been relied upon as the only mechanism for resolving conflict among coauthors. For example, coauthors should discuss openly and transparently who they believe is an author and in what order author names should be listed. The senior (and usually, corresponding) author should apply relevant domain-specific practices and be fair and consistent in their judgment. Involved parties can be expanded to provide different perspectives and partners, including peers, supervisors, and department leaders. Most institutions, including the NIH, have an Ombuds Office (https://ombuds.nih.gov/), which supports disputants to voluntarily work together to resolve potential conflicts. Other offices in the IRP, such as the Office of Intramural Training & Education (https://www.training.nih.gov/), the NIH Agency Intramural Research Integrity Office (https://oir.nih.gov/about/leadership-staff) or the HHS Office of Research Integrity (https://ori.hhs.gov/), can provide educational resources and/or sometimes advise disputants on how to effectively advocate for themselves or empathize in a dispute. The informal stage is an unstructured stage of negotiating, with decision-making that is managed by the senior and/or corresponding author(s) rather than by an institutional official, and for which that individual retains the authority to make a final decision about authorship, to which the coauthors typically “agree.” However, these informal methods can be hampered by disputants’ poor or incomplete understanding of authorship norms and practices. Some coauthors might lack the ability to be introspective and weigh their contributions fairly against the contributions of others (Bird, Hosseini, and Plemmons 2023, 44–46). Junior coauthors might lack the required vocabulary and assertiveness to articulate their position, or be excluded from authorship discussions. Moreover, counterproductive reactions, emotions, and positional negotiation strategies (e.g., negotiating on a fixed position rather than on overall interests that would allow compromises) can impede the informal process. Such challenges are more pronounced when authorship disputes reflect larger interpersonal conflicts among researchers, including with supervisors or collaborators. Either way, the informal process should not be extended beyond three months (unless all parties agree to an extension for further negotiations), and at any time during this window, any of the disputants may trigger the formal process, which is a relatively new and evolving process in which the institution becomes a party to the decision about authorship.

### The formal adjudication stage

The formal adjudication stage may be triggered for disputes over the assignment of authorship or the order of authors, on publications or presentations supported by the NIH IRP, if an author (first, last, middle, corresponding) or putative author is/was an NIH IRP researcher during the time the research project was active. The formal adjudication process is triggered by sending an e-mail to the institutional research integrity officer, who oversees the formal conflict resolution process through the following three stages: 1) fact-finding by a neutral party; 2) making a binding decision; and 3) institutional enforcement of the binding decision.

The factfinder can be a research integrity staff or someone in a leadership position in the NIH Institute in which the author(s) is employed. Factfinder(s) might consider the testimony from the disputants or other individuals with relevant information about the project, testimony from outside experts, and evidence from publications, presentations, drafts of manuscripts, research/lab records, research proposals, and e-mails. The factfinder may generate a report, with or without recommendations, which is reviewed and considered by the Deciding Official (DO), usually the Scientific Director of the Institute of the corresponding author(s). In cases where two or more institutes are involved, the DO is the NIH Deputy Director for Intramural Research. Upon examining the report, the DO makes a binding decision. Typically, the DO determines whether the authors’ names and order are correct as presented in the draft manuscript or if it should be modified in accordance with the request of the party who initiated the process. The determination means that the authors may proceed with submiting the paper to their target journal; or, if the paper has been published and requires correction, that the authors may request a correction. Sometimes the DO adds other conditions, such as sharing co-corresponding designation or adding a specific statement to the “Contributions” or “Acknowledgment” sections related to the contributions of one of the disputants. Failure to abide by the binding decision may result in action by the NIH, which could include contacting the journal to which a manuscript has been submitted to request a withdrawal, correction, or retraction. Binding decisions are supported by the NIH leadership if a journal requires further assurance of NIH’s commitment to the decision. In the case of researchers who might no longer be affiliated with the NIH, the DO will notify research integrity officials at those researchers’ current institution. Typically, journals rely upon institutional officials to resolve authorship issues, and therefore, it is unlikely that a disputant could bypass the institutional decision by working directly with a journal. The NIH makes every effort to work with extramural institutions that have coauthors impacted by the binding decision, as well as with the publishers of the contested paper, to ensure that the binding decision is faithfully executed. The IRP at NIH handles roughly a dozen new authorship conflicts per year. In roughly half of these cases, the coauthors seek assistance with informal resolution, while others seek formal adjudication. About 75% of all reported conflicts get resolved within a few months, in most cases leading to manuscript submission.

## Using innovative tools for factfinding

The DO’s ability to fairly and consistently determine a resolution to an authorship dispute depends, among other factors, on whether the factfinder has sufficient evidence of each coauthor’s relative contributions. Several tools can assist in factfinding. The first is the Contributor Role Taxonomy (CRediT). This community-owned resource consists of 14 standard contribution types (NISO 2025) that help specify individual contributions to publications (Table 1). Currently, some publishers (e.g., PLOS, see: https://journals.plos.org/plosone/s/authorship) mandate using CRediT as part of their submission workflow and others only recommend using it to disclose contributions without mandating it (e.g., Sage, see: https://www.sagepub.com/journals/information-for-authors/submitting-your-manuscript/credit). It should be noted that not every project should have all 14 contributor roles, and an individual can receive more than one role.

Within the NIH IRP, the CRediT taxonomy has proven an effective resource for the factfinding process in authorship disputes because it requires disputants to adopt a similar vocabulary for discussing contributions. Within the factfinding process at the NIH IRP, disputants are provided a spreadsheet with a list of the 14 roles, and asked to indicate the contributions of each involved researcher to the disputed manuscript. The spreadsheet includes both the title and the definition of each role to prevent miscommunication and misunderstanding, and includes a column titled “Additional Notes” to add other important caveats and nuances. This might be the first time disputants articulate their involved contributions using common terms and in a systematic manner. Within the NIH IRP, the roles of “Conceptualization,” “Investigation,” “Project administration,” “Supervision,” and “Writing – original draft” are frequently cited as roles with a major impact on authorship or authorship order. Our experience suggests that utilizing the CRediT taxonomy not only supports resolving a dispute, but it also encourages research groups to use it for documenting and assessing contributions in the future.

A second tool that NIH developed is specifically related to the “Investigation” role in the CRediT taxonomy (defined as: Conducting a research and investigation process, specifically performing the experiments, or data/evidence collection). The reason for isolating this role is that for the NIH IRP disputes, data acquisitions, analyses, and interpretations that result in the generation of new figures are among consistent determinants of authorship and authorship order. A common assumption within this context is that the more figures one contributes to, and the higher the impact of that figure on the overall message of the paper, the more prominent an authorship position that contributor should have. To better determine each coauthor’s role in investigations, disputants are provided a blank spreadsheet that lists each figure in the disputed manuscript, and are asked to indicate which coauthors contributed to data acquisition, analysis, and/or interpretation, and what their contributions were. Using this tool, it is possible to get a semi-quantitative assessment of which coauthors are responsible for the generation of the data for which figures, which can be very instructive for the factfinder and DO of the authorship dispute. Depending on whose data is questioned as being significant for the manuscript, disputants, and coauthors are asked to provide the dates of acquisition of the primary data presented in each data panel. Details about dates are important because sometimes disputes can arise when a coauthor leaves the institution, and others make major contributions to the work afterward. For example, a departed co-author might have had a significant role in conceptualizing a project and establishing the experimental design, but these contributions might likely get outweighed by the contributions of others who further collected, analyzed, interpretated and visualized the data, and subsequently wrote the first draft of the manuscript.

## Discussions and conclusions

Some disputants might welcome a specific numeric or percentage-based system for determining authorship and/or authorship order. Our experiences accord with Hosseini (2021) who has argued that developing a numeric scoring rubric for authorship claims is unfeasible because scholarly contributions are nuanced and context-specific. However, using semi-quantitative tools, such as those discussed in the previous section, can help a reasonable person to discern relative contributions to resolve a dispute. This information does not replace interviews or investigations of manuscript drafts and lab notes, but certainly enhances the factfinders’ understanding of contributions. In many cases, senior investigators agree on general principles (i.e., credit and accountability) around authorship and authorship order, essentially adopting the recommendations provided in Guidance and Policies for the Conduct of Research (Committee on Scientific Conduct and Ethics – CSCE 2023a), but are not necessarily able to motivate or articulate their decision to the research team or an unhappy disputant. The use of suggested tools allows researchers to see whether and how their authorship decisions are supported by standard contribution categories.

We anticipate that when groups are discussing authorship disputes, using tools like CRediT to parse contributions might lead to more consistent and harmonious decisions. These improvements could result in longer-lasting benefits for collaborative research environments. Future research can test this hypothesis, for example, by collecting and analyzing anonymized data about authorship disputes and the impact of employing these tools. CRediT has been useful in resolving authorship disputes at the NIH IRP, but other contributor taxonomies might be more appropriate in settings that have unique tasks and contribution types not captured by CRediT.

One limitation of using CRediT to resolve authorship disputes is that while authorship lists (that are not ordered randomly or alphabetically) specify the author who contributed the most (i.e., the first author) and the author responsible for the overall integrity of the work (e.g., the last or corresponding author), CRediT cannot always be useful when thinking about these issues.

With regard to disputes about who contributed the most (and subsequently, authorship order), contributing to more unique roles does not necessarily imply a more significant contribution to the overall success of a project. Specific tasks like data collection might sometimes require significant effort to complete, while roles like “Conceptualization,” “Methodology,” “Project administration” and “Writing – Review and editing” might appear to take less effort. However, it is the responsibility of the corresponding author to determine the relative significance of the contribution, which may or may not be related to the time it took to complete a specific task. In other words, there is no linear relationship between the time it took to complete a task and its relative significance for the project/publication. A very important prerequisite to conducting tasks like “Conceptualization” and “Methodology” is experience. However, experience can neither be quantified nor replicated.

While some disputants might be interested in specifying one task (conducted by the disputant) as the most significant one in a project, CRediT would not help in such determinations because the 14 roles are not inherently hierarchical and provide no tiered indication for significance. Research involves a tangled web of interrelated roles that depend on each other and affect the overall quality and originality of a publication. Sometimes disputants might be interested in interpreting the significance of a task in terms of its contribution to the most novel/original aspect of a research project, and other times in terms of enabling a host of other tasks, such as tasks that happen at the beginning of the research process (e.g., ”Conceptualization” or ”Funding acquisition”). Here too, CRediT cannot be helpful because again, CRediT is a nonhierarchical taxonomy.

With regard to disputes about responsibility for the overall integrity of the work, although CRediT has a “Supervision” role (which almost describes what the author responsible for the overall integrity of the work would do), because there are no specific guidance and norms about how to assign this role, different groups and individuals might interpret, and employ it differently. As a result, requesting that disputants contextualize contributions and provide as much detail as possible (e.g., dates when someone has been involved, unique expertise they brought to the group, which researchers were directly involved in supervising junior researchers, etc.) would be necessary.

Addressing authorship disputes can be time-consuming and stressful for all coauthors. Administrators, those engaged in Responsible Conduct of Research programs, and research integrity officers at the NIH IRP have found that a clear and consistent authorship dispute resolution process can de-escalate the tensions between authors, and lead to a resolution more quickly and amicably, leaving relationships intact for future collaborations. Tools used to resolve authorship disputes might also be helpful as a resource that enables research groups to better navigate future authorship conflicts.

## Figures and Tables

**Table 1.** CRediT contributor roles.

Roles	Description
Conceptualization	Ideas; formulation or evolution of overarching research goals and aims.
Data curation	Management activities to annotate (produce metadata), scrub data and maintain research data (including software code, where it is necessary for interpreting the data itself) for initial use and later re-use.
Formal analysis	Application of statistical, mathematical, computational, or other formal techniques to analyze or synthesize study data.
Funding acquisition	Acquisition of the financial support for the project leading to this publication.
Investigation	Conducting a research and investigation process, specifically performing the experiments, or data/evidence collection.
Methodology	Development or design of methodology; creation of models.
Project administration	Management and coordination responsibility for the research activity planning and execution.
Resources	Provision of study materials, reagents, materials, patients, laboratory samples, animals, instrumentation, computing resources, or other analysis tools.
Software	Programming, software development; designing computer programs; implementation of the computer code and supporting algorithms; testing of existing code components.
Supervision	Oversight and leadership responsibility for the research activity planning and execution, including mentorship external to the core team.
Validation	Verification, whether as a part of the activity or separate, of the overall replication/reproducibility of results/experiments and other research outputs.
Visualization	Preparation, creation and/or presentation of the published work, specifically visualization/data presentation.
Writing – original draft	Preparation, creation and/or presentation of the published work, specifically writing the initial draft (including substantive translation).
Writing – review & editing	Preparation, creation and/or presentation of the published work by those from the original research group, specifically critical review, commentary or revision – including pre- or post-publication stages.
